# Pre-Pulse Inhibition of an escape response in adult fruit fly, *Drosophila melanogaster*

**DOI:** 10.21203/rs.3.rs-3853873/v1

**Published:** 2024-01-23

**Authors:** Erika Viragh, Lenke Asztalos, Michaela Fenckova, Tamas Szlanka, Zoltan Gyorgypal, Karoly Kovacs, Joanna IntHout, Pavel Cizek, Mihaly Konda, Emanuela Szucs, Agnes Zvara, Judit Biro, Eniko Csapo, Tamas Lukacsovich, Zoltan Hegedus, Laszlo Puskas, Annette Schenck, Zoltan Asztalos

**Affiliations:** 1 Institute of Biochemistry, HUN-REN Biological Research Centre, Szeged, Hungary; 2 Aktogen Hungary Ltd., Szeged, Hungary; 3 Aktogen Ltd., Department of Genetics, University of Cambridge, Cambridge, United Kingdom; Current address: Aktogen Ltd. Ramsey, Huntingdon, United Kingdom; 4 Department of Human Genetics, Donders Institute for Brain, Cognition and Behaviour, Radboud University Medical Center, Nijmegen, the Netherlands; 5 Department of Molecular Biology and Genetics, Faculty of Science, University of South Bohemia in Ceske Budejovice, Ceske Budejovice, Czechia; 6 Institute of Biophysics & Core Facilities, HUN-REN Biological Research Centre, Szeged, Hungary; 7 HCEMM-BRC Metabolic Systems Biology Lab, Szeged, Hungary; 8 Department for Health Evidence (HEV), Radboud University Medical Center, Nijmegen, The Netherlands; 9 Voalaz Ltd., Szeged, Hungary; 10 Laboratory of Functional Genomics, HUN-REN Biological Research Centre Szeged, Hungary; 11 University of Zurich, Brain Research Institute, Zurich, Switzerland

**Keywords:** Pre-Pulse Inhibition, *Drosophila*, 3Rs, behaviour system, *Nmdar1*, *Dysbindin*

## Abstract

Pre-Pulse Inhibition (PPI) is a neural process where suppression of a startle response is elicited by preceding the startling stimulus (Pulse) with a weak, non-startling one (Pre-Pulse). Defective PPI is widely employed as a behavioural endophenotype in humans and mammalian disorder-relevant models for neuropsychiatric disorders. We have developed a user-friendly, semi-automated, high-throughput-compatible *Drosophila* light-off jump response PPI paradigm, with which we demonstrate that PPI, with similar parameters measured in mammals, exists in adults of this model organism. We report that *Drosophila* PPI is affected by reduced expression of *Dysbindin* and both reduced and increased expression of *Nmdar1* (N-methyl-D-aspartate receptor 1), perturbations associated with schizophrenia. Studying the biology of PPI in an organism that offers a plethora of genetic tools and a complex and well characterized connectome will greatly facilitate our efforts to gain deeper insight into the aetiology of human mental disorders, while reducing the need for mammalian models.

An estimated one in three people report mental health problems at some point in their lives. This makes mental disorders one of the biggest health burdens on individuals, families and societies^1^. The employment of biological model systems in basic science and pre-clinical studies for treatment development is inevitable. As there are no reliable biochemical or cellular tests for mental disorders, these model systems must include intact animals^2^.

It is important to establish common intermediate phenotypes or biomarkers, in genetic epidemiology and psychiatric genetics commonly referred to as endophenotypes, to create a link between the experimental measures in animal models and human individuals. Pre- Pre-Pulse Inhibition (PPI) is a neural process where suppression of a startle response is elicited by preceding the startling stimulus (Pulse) with a weak, non-startling one (Pre-Pulse). PPI is considered to be an operational measure of sensorimotor gating, or the ability of a sensory event to modulate a motor response^3^. Measuring PPI is used to identify deficits in early-stage information processing. Impaired PPI is primarily reported in schizophrenic individuals but also in individuals with other psychiatric disorders^4^. PPI performance is likely an indicator of basic aspects of inhibitory neural processes. PPI and similar indicators are amenable to be studied in cross-species models relevant to these disorders. Abnormal PPI is extensively used as a behavioural endophenotype in humans and disorder-relevant mammalian models, and reveals neuropsychiatric disorder states and potential antipsychotic drug effects^4,5^.

Although, the results obtained in vertebrate disease models, most commonly rat^6,7^ (Rattus norvegicus domesticus), mouse^8,9^ (Mus musculus) and zebrafish larva^10,11^ (Danio rerio) significantly enriched our knowledge, the employment of these animals raises ethical concerns. For this reason, there are efforts made to replace them with invertebrates, where PPI has also been described.

In adults of the common locust species, *Locusta migratoria* a jerky startle movement to a loud sound can be inhibited with a preceding quieter noise^12^. The sea slug, *Tritonia Diomedea,* offers its large neurons for describing multicellular mechanisms of PPI of its stereotypic escape response^13,14^. Nevertheless, these two organisms allow for only limited genetic interventions to be carried out, which hampers progress in delineating the molecular and genetic components of PPI. In the nematode *C. elegans*, a model with characterized connectome amenable to genetic manipulation, only startle inhibition, but not PPI was demonstrated^15,16^.

Research in the fruit fly *Drosophila* has already contributed to uncovering biological mechanisms underlying psychiatric disorders^17–20^. However, the lack of quantifiable PPI was identified to hamper progress in this field^19^.

In *Drosophila* a very sophisticated set of genetic tools are available and continue to expand. These tools include connectome databases^21–23^, genetic methods for generating targeted mutants^24,25^ and for neural circuit-specific manipulation^26,27^, moreover neural activity detection and optogenetic toolkits^28^.

The PPI phenomenon in *Drosophila* was first described in larva, using a startle response to the buzz of a wasp predator^29,30^. Even though the behaviour paradigm is robust enough to show effects for RNA interference (RNAi) or mutations in genes associated with human mental disorders, it has not been adapted to perform experiments in high throughput.

It has been previously observed both at electrophysiological and behavioural levels that the adult *Drosophila* escape/startle response, the light-off jump response, can be habituated^31^. In these light-off jump response habituation behaviour experiments single *Drosophila* were observed by an experimenter and the presence of jump responses was noted manually. To improve this system, we have developed a semi-automated method for the detection of *Drosophila* jump responses, which allows precise control of experimental variables and relatively high throughput. In this behaviour paradigm it is now feasible to conduct large-scale genetic or compound screens that affect habituation^32^.

We report here the discovery that the light-off jump response can also be suppressed by a preceding dimming of light, expanding the applicability of this behaviour to measure Pre-Pulse Inhibition properties in adult *Drosophila*.

To validate our PPI paradigm in the neuropsychiatric field we tested *Drosophila* adults in which we manipulated the orthologues of two established human schizophrenia susceptibility genes. In our “schizophrenia models” *Nmdar1*^33–37^ (*N-methyl-D-aspartate receptor 1*), an orthologue of *GRIN1* was either down- or up-regulated, while *Dysbindin*^38–41^, an ortholog of *DNTBP1* was down-regulated.

NMDAR is one of three types of ionotropic glutamate receptors in vertebrate neurons and it has an important role in synaptic plasticity and learning & memory functions^42^. NMDA receptor defects can lead to nervous system disorders^43^, and there is a well-supported “glutamate hypothesis” of schizophrenia^44,45^. NMDA receptors exist in the *Drosophila* nervous system, as well, and play an important part in learning & memory processes^33,34,36,37^. In accordance with this evolutionary conservation, we were able to detect decreased PPI performance both in knock-down and overexpression of *Drosophila Nmdar1* in our PPI system (see Results).

*Dysbindin-1* (dystrobrevin-binding protein 1, encoded by *DTNBP1*) was identified as a constituent of the BLOC-1 (biogenesis of lysosome-related organelles complex 1) and DPC (dystrophin-associated protein complex) complexes, which are involved in maintaining the structure of muscle as well as neuronal synaptic membrane and in endosomal trafficking, neurotransmitter release as well as neural development, respectively^46–50^.

Mutations in the *DNTBP1* gene or reduction in its mRNA and protein levels are associated with a higher occurrence of schizophrenia^51–53^. Rodent and *Drosophila* models of *DNTBP1* defects successfully recapitulated some of the schizophrenia hallmarks and phenotypes, such as changes in neurotransmitter release and impaired social behaviour, as well as cognitive deficits, and contributed significantly to the understanding of schizophrenia aetiology^38–41,53,54^ In our new behaviour paradigm, knock-down of *Drosophila Dysbindin* was also characterized by PPI defects.

Based on these findings the use of the *Drosophila* PPI behavioural paradigm presented here permits experiments that up to date have been unfeasible in mammalian/rodent disorder-related models and are characterized by much lower cost and higher efficiency. The PPI paradigm established has the potential to fundamentally contribute to the understanding of molecular, genetic and neural processes underpinning mental capacities, their deficits in disorders and open up avenues to novel treatment strategies.

## Results

### PPI-enabled Light-off Jump Response System

We have previously developed a semi-automated, high-throughput behaviour system to measure neural processes in the visual sensory domain of the fruit fly. An earlier version of this system to measure habituation is already in use and some of the system’s basic properties, such as that the jumps are specific responses to the light-off stimuli and that the jump detection is accurately coupled to wing vibration have already been described^32^.

**Fig. 1:**
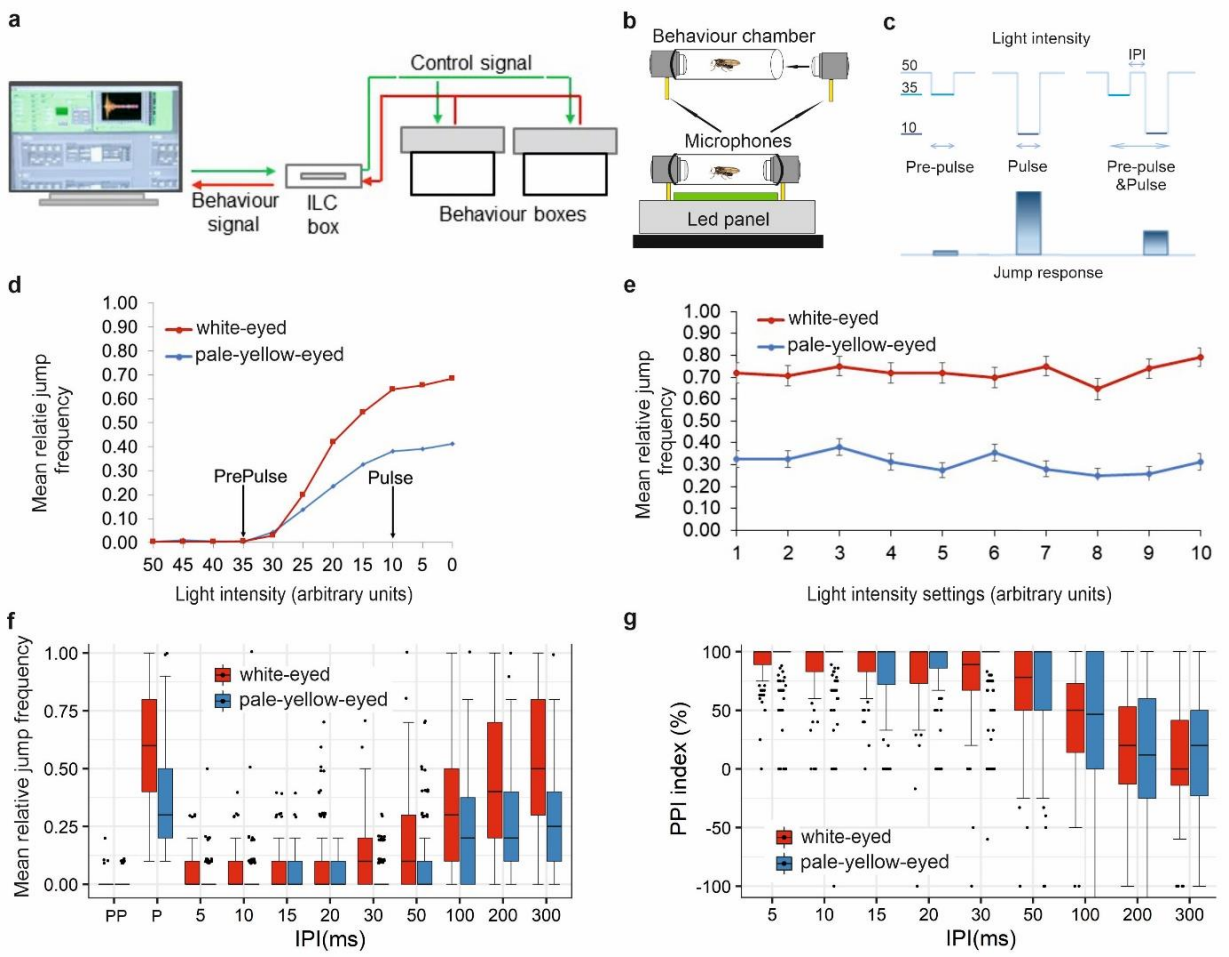
Light-off jump response system for measuring Pre-Pulse Inhibition in *Drosophila*. **(a)** Schematic representation of Light-off jump response system, (ILC: Intelligent Light Controller). **(b)** One individual fly is tested in each behaviour chamber. **(c)** Elements of the Light-off jump response Pre-Pulse Inhibition protocol: weak [Pre-Pulse (PP), 50→35], strong [Pulse (P), 50→10] or combined weak-and-strong [PPI), 50→35 & 50→10] light dimming stimuli separated by Inter Pulse Interval (IPI). All light intensity values given here and subsequently are in arbitrary light units, except otherwise stated. **(d)** Jump response of white-eyed (*w*^*1118*^, n=96) and pale-yellow-eyed (*GMR-wIR*, n=160) *Drosophila* to different light dimming stimuli (Initial light intensity 50, Dimmed light intensity range 50 to 0). “Pre-Pulse” and “Pulse” arrows represent the two chosen light intensity values used in subsequent PPI experiments. **(e)** Repeats of light-off stimuli at 5 s ITI evokes non-habituating jump responses in white-eyed (n=96) and pale-yellow-eyed (n=160) flies (50→10 light units, 15 ms duration). Light-off stimuli were repeated 10 times for the flies and the jump responses were averaged for each successive repeats (1 to 10 on X axis) for all flies. Experiments were conducted on three or five different days. **(f)** Pre-Pulse Inhibition at different IPI durations. Relative Jump frequencies of white-eyed (n=160) and pale-yellow-eyed (n=160) flies to PPI light dimming stimuli (described in **d)** at 5 to 300 ms IPIs are represented by box plots. Experiments were performed on five different days. **(g)** *Drosophila* PPI expressed in PPI indices. PPI indices are calculated from jump response data shown in panel **f** by the formula: 100 – ((PPI score/Pulse score) x100)^55^. For full genotypes see Supplementary Table 1.

We adapted this system to assess PPI (also see Methods). This device (Fig. 1a,b; and Supplementary Fig. 1) now is capable of switching off and/or dimming light in a finely controlled and fully automated manner to evoke an innate jump-and-flight startle response. The main technical improvement is the capacity to deliver a complex “Pre-Pulse & Pulse” compound light stimuli for the PPI protocol (Fig. 1c).

### Optimalization of the Pulse and Pre-Pulse stimuli for PPI

The light-off jump response depends on the eye colour of the flies ^31,56–58^. Available genome-wide collections of *Drosophila* transgenic RNAi strains as well as mutants have a wide-range of eye colours, which would hamper large-scale screening and comparison of manipulated genotypes (Supplementary Fig. 2). For this reason, we aimed to find optimal light-off/dimming stimuli used in PPI experiments for different eye-coloured flies.

Indeed, white-eyed (*w*^*1118*^) flies gave stronger jump reactions than pale-yellow-eyed (*GMR-wIR*, i.e., eye-specific *white* RNAi knock-down^59,60^) flies (Fig. 1d,e; – for full description of genotypes see Supplementary Table 1), while flies with wild-type eye colour do not produce experimentally informative jump responses to these light-off/dimming stimuli^31,56–58^.

When we exposed flies to a series of complete light-off stimuli starting from different Initial light-intensity values, strong jump responses were observed both in white-eyed and in pale-yellow-eyed flies. Although the two strains show different maximum jump frequencies, they reach the “Initial light intensity vs. Jump response” curve’s asymptotes at very similar Initial light intensity values (Supplementary Fig. 3). Based on these results we chose 6.36 lux (corresponding to 50 arbitrary light units of the behaviour system, Supplementary Fig. 4) as default Initial light intensity for further light-off/dimming jump response experiments (see arrow in Supplementary Fig. 3). This Initial light intensity value evokes maximum response at the lowest possible light intensity so that overstimulation the visual system during the experiments could be avoided.

Next, we looked for an efficient Pre-Pulse stimulus that can inhibit the response to an appropriate Pulse stimulus in PPI experiments. When flies were exposed to light intensity reduction, i.e., dimming, jump responses still were observed. In dimming, Initial light intensity 50 was dimmed to X, i.e., “Dimmed light intensity”, where X>0 (light dimming 50➔X, Fig. 1d). From the resulting “Light-dimming vs. Jump response” curve, two X values for the dimming stimuli were selected, one for Pre-Pulse and one for Pulse. At 50➔35 dimming, the flies produced infrequent jump responses, which showed that they were able to perceive the stimulus but the negligible response to it does not interfere with the Pre-Pulse Inhibition results. In PPI experiments we used this 50➔35 dimming as Pre-Pulse stimulus. For Pulse we chose the stimulus 50➔10 dimming, to which flies gave a strong jump response, comparable to the maximum response to a complete light-off stimulus (50➔0), but potentially easier to suppress by the Pre-Pulse (Fig. 1d).

When complete light-off stimuli are frequently repeated (e.g., every 1 second, i.e., 1 s Inter Trial Interval – ITI) jump response habituation. i.e., response decrement, upon repeated stimulus presentation occurs (Supplementary Fig. 5a,b). Measurements of this non-associative learning have already been extensively employed in cognitive disorder studies^32,61–63^. When the light-off stimuli were presented less frequently, no influence of the previous stimulus on the response (i.e., no response decrement) was detected, and at 5 second ITI, the subsequent trials can be considered as independent ones (Fig. 1e). Consequently, 5 s ITI can be used for repeat trials in PPI experiments.

Taken together we established parameters for a Light-off jump reflex Pre-Pulse Inhibition protocol (Fig. 1c), in which a weak Pre-Pulse (50^35) dimming stimulus can be followed by a strong Pulse (50➔10) dimming stimulus separated by various durations of Inter Pulse Intervals (IPIs, to be determined later). These complex PPI stimuli can be presented in 5 s Inter Trial Intervals in random order to measure independent jump responses to them.

### Light-off jump response PPI manifests in adult *Drosophila*

The optimized Pre-Pulse and Pulse light-dimming stimuli and their temporal relationship is depicted in Fig. 1c. To establish optimal IPI for a Pre-Pulse to suppress jump response to a subsequent Pulse stimulus in a combined PPI stimulus we tested the effect of a range of biologically relevant Inter Pulse Intervals (IPI, 5-1,000 ms) on jump response frequency^64,65^. The results clearly demonstrated that adult *Drosophila* show Pre-Pulse Inhibition in the range of 5-200 ms IPIs (Fig. 1f). The results revealed that the smallest jump frequency to PPI stimuli was detected at IPI 5-50 ms, i.e., the strongest PPI was detected in this IPI range. The jump frequency gradually increased at longer IPIs, reaching the same jump frequency as to jump frequency to Pulse alone at IPI 300 ms, meaning that the PPI performance gradually waned from IPI 100 to 300 ms (Fig. 1f). At even longer IPIs (400-1,000 ms) neither PPI nor Pre-Pulse Facilitation was observed (Supplementary Fig. 6). The results also showed that although white-eyed and pale-yellow-eyed flies jump with different frequency to the Pulse stimuli (Fig. 1f), they exhibit the same PPI characteristics (Fig. 1f,g and Supplementary Fig. 7).

Here we demonstrated light-off jump response Pre-Pulse Inhibition in adult *Drosophila* for the first time that exhibits similar properties to those of higher organisms.

### Analysis of light-off jump response PPI data

The canonical measure of PPI is the PPI Index^55^, calculated as a percentage reduction of the response to the Pulse stimulus:

$$\mathrm{PPI}\;\mathrm{Index}\left(\%\right)=100-\left(\frac{\mathrm{PPI}\;\mathrm{score}}{\mathrm{Pulse}\;\mathrm{score}}\times 100\right)$$

PPIIndex(%)=100%=CompletePre−PulseInhibition

PPIIndex(%)=0%=NoPre−PulseInhibition

The PPI scores expressed as Relative jump frequency (referred to as Jump frequency, too) in Fig. 1f are also presented in PPI Indices in Fig. 1g and Supplementary Fig. 8b.

In the literature PPI result calculations are based on the assumption that the startle response has an approximately Gaussian distribution^10,15,65–67^. This assumption found to be false in rats where the startle response distribution is better represented by a log-normal distribution^68^. However, our startle data showed binomial distribution (0 or 1 jump), therefore we employ a logistic regression model to analyse the effect of the preceding Pre-Pulse on the jump response to Pulse.

### Logistic regression analysis of PPI

**Fig. 2:**
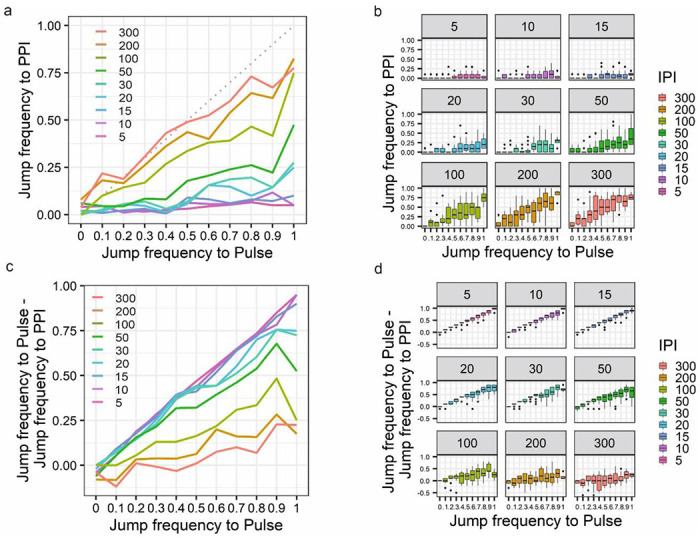
Dependency of the Jump response to PPI stimuli on the response to Pulse stimuli. **(a)** Line plot of the mean Jump frequency to PPI as a function of Jump frequency to Pulse stimuli at different Inter Pulse Intervals. **(b)** Boxplot showing the distributions of the jump frequency measurements underlying the mean frequencies shown in **a. (c)** Line plot of Jump frequency to PPI minus Jump frequency to Pulse as a function of Jump frequency to Pulse stimuli at different Inter Pulse Intervals. **(d)** Boxplot representation of the results shown in **c.** Data were collected over two years with white-eyed control flies (n=1,512).

In our experiments a group of flies typically contains individuals with jump frequency ranging from 1 (10 jumps in 10 Pulse trials) to 0 (0 jumps in 10 Pulse trials). We therefore asked whether this could have an influence on the PPI jump response and subsequent analysis. We sub-grouped a set of control flies into categories based on their jump frequency to Pulse stimuli and plotted their jump frequency to PPI stimuli in all tested Inter IPIs. We observed that flies with low jump response to Pulse stimuli do not show as efficient suppression of jump response to PPI stimuli as flies with higher jump response to Pulse stimuli (Fig. 2a,b). This effect is better visualized by plotting the jump frequency data as Pulse vs Pulse-minus-PPI (Fig. 2c,d). The higher the difference, the better the suppression.

From these we concluded that the reduction in jump response to PPI stimuli – i.e., the manifestation of PPI – is confounded by the original capacity of the fly’s jump response to the Pulse stimuli, and therefore should be included in the analysis. This statistical approach allows us to include the low jumpers in the analysis as they bear valuable information, too. They show PPI, albeit their PPI response has smaller resolution.

Because of the binary character of the jump response data, we applied a logistic regression model using a generalized linear mixed-effects model (GLMM) framework^69^ in order to assess the effect of a variable of interest, e.g. genotype, on jump response following Pre-Pulse, Pulse or their combination. This framework allowed us to control for any bias caused by the experimental design, such as, individual flies, testing day, and behaviour system, which were included in the model as random effect variables (also see Methods). In order to control for the Pulse-PPI correlation, we also included in the model estimating the response to PPI stimuli an interaction term between response to Pulse and PPI stimuli at different IPIs (Methods).

**Fig. 3:**
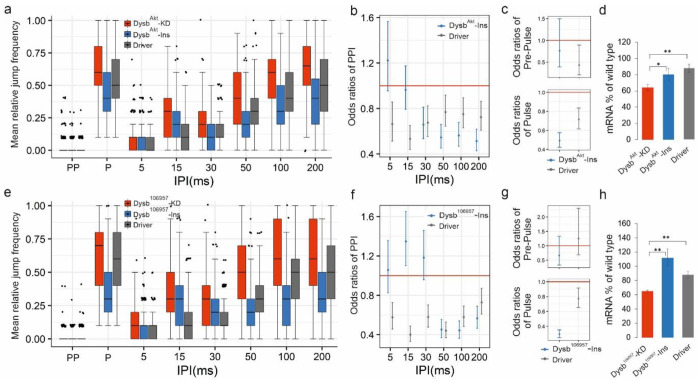
Knock-down mutants of schizophrenia susceptibility gene orthologue *Dysbindin* show PPI phenotype. **(a** and **e)** Jump frequency of *Dysbindin* RNAi Knock-down *flies* (*Dysb*^*Akt*^-KD, n=242 and *Dysb*^106957^-KD, n=248, respectively) compared to the appropriate control genotypes, *UAS-Dysb-RNAi* inserts alone (*Dysb*^*Akt*^-Ins, n=235 and *Dysb*^*106957*^-Ins, n=230, respectively), as well as *2xGMR-wIR; elav-Gal4, UAS-Dicer-2* (Driver, n=248 and n=253, respectively) alone in PPI experiments. For detailed genotypes see Supplementary Table 1. **(b,c** and **f,g)** Control/mutant jumping odds ratios (genotypes as in **a** and **e**) following PPI, Pre-Pulse as well as Pulse stimuli estimated by GLMM. At odds ratio 1 the red horizontal line indicates equal odds of jumping for a mutant (*Dysb*^*Akt*^-KD or *Dysb*^106957^-KD) and a given control genotype. Odds ratios for the two control genotypes are calculated as control/mutant jumping odds separately. Therefore, for a given control genotype odds ratios below 1 indicate lower chance of jumping, and in the case of PPI stimuli, stronger PPI compared to the mutant (i.e., the mutant PPI is weaker). When the error bars representing the 95% confidence intervals do not overlap with the odds ratio=1 red horizontal line, it is considered to be a sign of statistically significant difference at the 0.05 significance level. **(d** and **h)** Relative levels of *Dysb* mRNA, as determined by qPCR, in indicated genotypes. *: P< 0.05, **: P< 0.01.

Jump response differences between two genotypes are estimated as odds ratios (e.g., see Fig. 3b,f). For a given genotype the odds of jumping is the frequency of jumping divided by the frequency of not jumping as a response to a given stimulus. The odds ratio is the ratio of these odds values of the two genotypes (mutant/control or control/mutant; also see Methods). With this model we found that n=128 sample size per group (new 32 flies tested on 4 different days) is sufficient for reproducible results (Supplementary Fig. 8).

### Testing mutants in schizophrenia susceptibility genes for PPI defects

In neuropsychiatry, abnormal PPI is a widely accepted biomarker in humans and mammalian models as PPI measurements can reveal disorder states^4^. Hence, we asked whether perturbing *Drosophila* orthologues of human schizophrenia susceptibility genes would affect light-off jump response PPI. For this, we used the UAS-Gal4 binary system^70^ in combination with the panneuronally expressed *elav-Gal4* driver to knock-down *Dysbindin*^41^ and *Nmdar1*^34^ by inducible RNAi. Our Gal4 driver line (Driver) also carried two additional genetic elements: *2xGMR-wIR* to render the flies white-eyed^61,62^) and *UAS-Dicer-2* to enhance the effectiveness of RNAi^71^. Consequently, experimental flies contained *elav-Gal4, UAS*—gene-of-interest—*RNAi, 2xGMR-wIR* & *UAS-Dicer-2* (for full description of genotypes see Supplementary Table 1). These flies were compared to *“UAS*—gene-of-interest—*RNAi* insert alone” and “Driver alone” Controls. We confirmed RNA silencing by measuring mRNA levels of the targeted genes by qPCR.

### Knockdown of *Dysbindin* causes a PPI defect in *Drosophila*

Knocking down *Drosophila Dysbindin* gene with RNAi construct *Dysb*^*Akt*^ showed significantly reduced PPI at IPI range of 30-200 ms (Fig. 3a,b). Knock-down with a second, independent and overlapping, RNAi construct *Dysb*^*106957*^ (for genotype see Supplementary Table 1) generated consistent results of reduced PPI at IPIs 50-200 ms (Fig. 3e,f). Furthermore, we observed increased jump frequency to the Pulse stimuli in both cases of *Dysb*^*Akt*^-KD and *Dysb*^*106957*^-KD (Fig. 3c,g lower panels). For GLMM-calculated p values of statistical significance see Supplementary Table 2. The PPI phenotype correlated well with the significantly reduced mRNA levels of *Dysb* in the knock-down flies (about 60% of wild-type with both RNAi constructs; Fig. 3d,h). For RNA levels, p values of statistical significance see Supplementary Table 3. In conclusion the *Dysbindin* downregulation is characterized by PPI defect and “hyper-reactivity” phenotypes.

### Knockdown of *Nmdar1* causes a PPI defect in *Drosophila*

Considering the glutamate hypothesis of schizophrenia, we analysed the effect of modulating *Nmdar1* transcript levels on PPI in *Drosophila*. To knock down the *Drosophila Nmdar1* gene two *UAS-RNAi* constructs were used. In respect of *Nmdar1*^*37333*^*-KD* we observed reduced PPI phenotype in the range of 50-200 ms IPIs. Meanwhile, in *Nmdar1*^*41666*^*-KD* flies reduced PPI phenotype was observed at 5, 100 and 200 ms IPIs (Supplementary Fig. 9b,f; p values in Supplementary Table 2). This relatively weak PPI phenotype obtained at *Nmdar1* mRNA levels of 49% and 58%, respectively (Supplementary Fig. 9d,h; p values in Supplementary Table 3).

Nevertheless, we wanted to generate a *Nmdar1* disease model with stronger PPI phenotype. This could be achieved by introducing an extra *Nmdar1* deletion to further deplete the mRNA level in Nmdar1-KD flies. For this purpose, we chose to work with the 21-mer sort hairpin RNA expressing *Nmdar1* RNAi construct (*UAS-Nmdar1*^*41666*^, instead of the long RNAi sequence-containing *UAS-Nmdar1*^*37333*^ one) to avoid the necessity of using *UAS-Dicer-2* in flies that will harbour multiple genetic elements. Hence, we introduced a heterozygous *Nmdar1* deletion into the *Nmdar1*^*41666*^*-KD* background (for full genotypes see Supplementary Table 1).

**Fig. 4:**
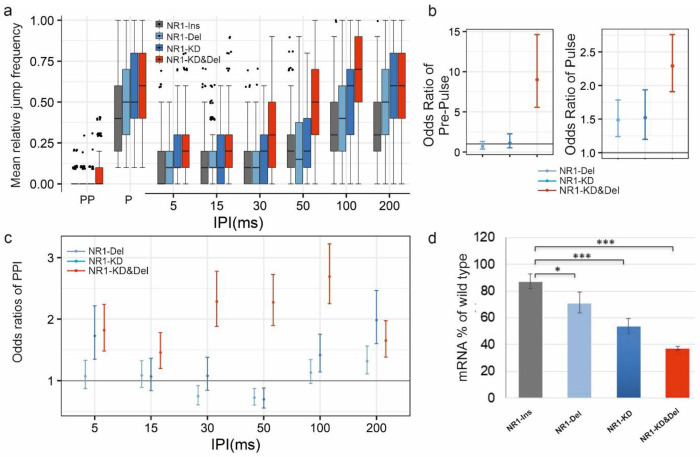
Down-regulation of *Nmdar1* (NR1) resulted in PPI phenotype. **(a)** Jump frequency of classical deletion (NR1-Del, n=258), RNAi Knock-down (NR1^41666^-KD, n=125) and combined NR1-KD&Del (n=253) mutants of *Nmdar1* compared to control, UAS-NR1^41666^ insertion alone, (NR1-Ins) in PPI experiments. For detailed genotypes see Supplementary Table 1 **(b)** Pre-Pulse and Pulse performances of *Nmdar1* mutants expressed in odds ratios estimated by GLMM. Odds ratios for each mutant genotype are calculated as mutant/control jumping odds separately. At odds ratio 1 the grey line represents equal jumping odds for a given mutant and the common control, >1 odds ratio indicates higher odds of jumping for the mutant compared to the control. 95% confidence intervals are shown. **(c)** PPI phenotypes of *Nmdar1* mutants expressed in odds ratios. At odds ratio 1 the grey line represents the given IPI having the same effect on the chance of jumping for control and mutant, >1 odds ratio meaning higher odds of jumping, thus weaker PPI for the mutant genotypes. **(d)** Relative levels of *Nmdar1* mRNA in the different genotypes. *: P< 0.05, **: P< 0.01, ***: P< 0.001.

Fig. 4 shows that Nmdar1-Deletion heterozygote flies (NR-Del), similarly to *Nmdar1*^*41666*^-*KD* (Nmdar1-KD) flies show weak PPI phenotypes, while the Nmdar1-KD&Del genetic combination results in a strongly reduced PPI phenotype at all IPIs (Fig. 4a,c). This is in accordance with the determined *Nmdar1* mRNA levels, where the lowest mRNA level (below 40%) was measured for the *Nmdar1*-Del&KD combination (Fig. 4d). We conclude that strong *Nmdar1* down-regulation causes severe PPI defect. Similarly to *Dysbindin* knock-down, an increased jump response to Pulse stimuli was observed in all the *Nmdar1*-KD, *Nmdar1*-Del and *Nmdar1*-KD&Del flies (Fig. 4b, right panel). In addition, *Nmdar1*-KD&Del flies respond more to Pre-Pulse stimuli than the control ones (Fig. 4b, left panel).

### *Nmdar1* overexpression causes a PPI defect in *Drosophila*

**Fig. 5:**
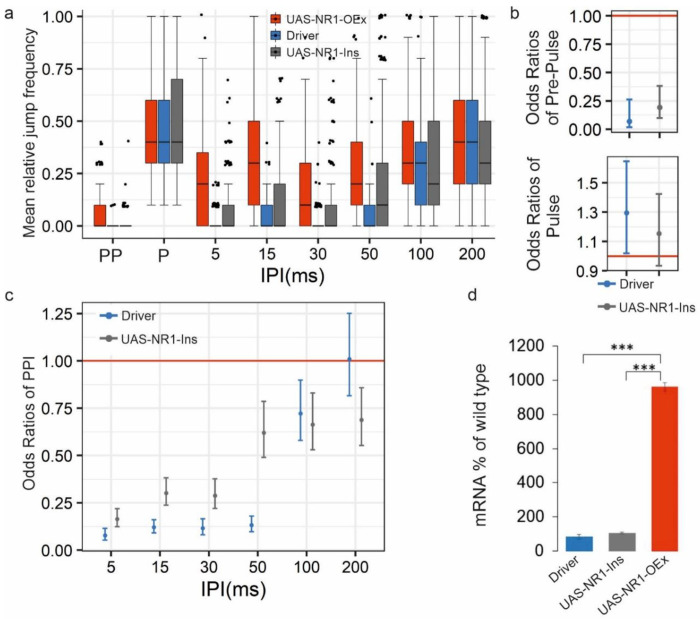
Up-regulation of *Nmdar1* (NR1) resulted in impaired PPI. **(a)** Jump frequency of full length NR1-cDNA neural overexpression transformant (UAS-NR1-OEx, n=175) compared to two control genotypes (UAS-NR1-Ins, n=177 and Driver, n=161) at different IPIs. **(b)** Pre-Pulse and Pulse performances presented in odds ratios. **(c)** PPI performances presented in odds ratios. Control/mutant jumping odds ratios following PPI, Pre-Pulse as well as Pulse stimuli estimated by GLMM. At odds ratio 1 the red horizontal line indicates equal odds of jumping for the mutant (UAS-NR1_OEx) and a given control genotype. Odds ratios for the two control genotypes are calculated as control/mutant jumping odds separately. Therefore, for a given control genotype odds ratios below 1 indicate lower chance of jumping, and in the case of PPI stimuli, stronger PPI compared to the mutant. The error bars represent the 95% confidence intervals. **(d)** Relative levels of *Nmdar1* mRNA in the different genotypes. *: P< 0.05, **: P< 0.01, ***: P< 0.001. For detailed genotypes see Supplementary Table 1.

We wanted to address the question if there is an optimal range of Nmdar1 mRNA level, required for PPI, by also overexpressing it in *Drosophila* ^35,72^. We expressed full-length Nmdar1-cDNA from a UAS construct driven by elav-Gal4. In these transformant flies the expression level of Nmdar1 mRNA was about 10-times higher than in wild-type flies (Fig. 5d).

The flies with this very high level of Nmdar1 expression showed impaired Pre-Pulse Inhibition at 5-100 ms IPIs (Fig. 5a,c). In this case higher jump frequency is observed for the Pre-Pulse stimuli, only (Fig. 5b, upper panel). In conclusion, similarly to knock-down, up-regulation of Nmdar1 resulted in impaired PPI, although in this latter case flies showed mild hyper-reactivity towards the Pre-Pulse stimulus.

## Discussion

Here we report a semi-automated, high-throughput light-off jump response behaviour paradigm for measuring Pre-Pulse Inhibition in adult *Drosophila*. One operator is able to simultaneously manage up to 32 single-fly tests in a 30-minute period. There are great advantages of this relatively high-throughput adult PPI measurement method in genetic or compound screens over the labour intensive, expensive and ethically questioned vertebrate (rodent and zebrafish) model based PPI methods. It is a general problem in measuring behaviour that the results are hard to compare due to the different and unique paradigms used to obtain them^19,68^. To enable the research community to measure *Drosophila* habituation and PPI in the same platform, Aktogen Limited, Cambridge, UK made this Light-off jump response system commercially available.

It was demonstrated that modelling neuropsychiatric disorders (NPDs) in flies can have both mechanistic and predictive validity^19^. As Pre-Pulse Inhibition is a widely used endophenotype of these disorders, our highly efficient *Drosophila* PPI paradigm can support applying the potentials of this genetically tractable model organism to better understand NPDs and develop effective treatments for them^19^.

### PPI manifests in adult *Drosophila*

In our behaviour system we demonstrate for the first time that the light-off jump response Pre-Pulse Inhibition (PPI) phenomenon exists in adult *Drosophila* and exhibits similar properties to those of higher organisms. PPI at Inter Pulse Intervals (IPI, also known as Lead Interval) in the range of 5-200 ms were observed, overlapping with the range of 30-500 ms in humans^64^ and the typical range of 2-500 ms in rodents^65^. These parallels in IPIs further suggest the usefulness of the adult *Drosophila* PPI system in reducing experiments on mammalian models. The high-throughput nature of our PPI method allows to test PPI at multiple IPIs in one experiment. This feature will allow more detailed descriptions of the effects of genetic mutations and/or chemical compounds on the underlying cognitive processes, with the potential to identify distinct molecular and/or neural mechanisms of these processes.

In *Drosophila* larval PPI, where there was no measurement below 100 ms IPI, the best PPI was detected at 300 ms IPI^29^. Meanwhile, in adults we could not measure PPI over 200 ms IPI. We speculate that the difference in the PPI characteristics in the two developmental stages originates from the way in which the nervous system processes information. Moreover, it may also matter that the startling/PPI stimuli travel differently in the two behaviour systems (light for the adult vs sound – possibly in the agar base – in case of the larval assay).

Recently, Schioth at al published a paper on PPI in adult *Drosophila*. In that study testing parameters are very different from what are generally considered as PPI experimental conditions^73^. Studying the details of their results we did not find the case for this PPI phenomenon convincing. As PPI is always measured on modulating startle response it is concerning that for the light-off stimulus they detected movement response with reaction time about 100x, and speed about 1,000x slower than the known *Drosophila* jump-and-flight startle reaction^31,57,74^. Even more alarmingly, the authors did not present data about the effect of the Pre-Pulse stimulus alone, which would change the reference activity in their calculations. If the Pre-Pulse stimulus evokes a reaction on its own the claimed PPI phenomenon would be put into question. Also, in their work the durations of the effective IPIs are about 10-1,000x longer than the typical IPI durations from mammalian^7,9,65,75^ or other PPI works^10–12,14^, including our own light-off jump response PPI.

### Individual differences in stimulus reactivity affect PPI

While analysing light-off jump response PPI data we noticed that the jump response of each individual fly to PPI stimuli was dependent on its jump response to Pulse stimuli, as a Pulse response is prerequisite for the Pre-Pulse to show suppression. This confounded a proper assessment of PPI performance by the conventional PPI index, which is in accordance with earlier findings in human and mouse experiments^76^ and recently published results obtained in rats^68^. To circumvent this problem we applied a logistic regression model using a generalized linear mixed-effects model (GLMM) framework^69^ on the PPI data. This model was designed to control for potential confounding factors on PPI such as i) jump response to Pulse alone, ii) batch effects caused by the experimental design, iii) individual differences between flies. We propose to apply this approach to the analysis of *Drosophila* PPI data with a binomial distribution (0 or 1 jump in this case). Furthermore, we propose that the same approach might be valuable in the analysis of PPI data with different distributions, where there are also needs to correct for effects of experimental design and individual differences.

### Testing mutants in schizophrenia susceptibility genes for PPI defects

We show that our light-off behaviour system can monitor changes in PPI performances in genetically manipulated *Drosophila*. We were able to detect PPI phenotypes by decreasing or increasing the expression of two *Drosophila* orthologues of human schizophrenia susceptibility genes (*Dysbindin*^38–41^ and *Nmdar1*^33,34,36,37^). Panneural down-regulation of *Dysbindin* and *Nmdar1* transcription resulted in a reduced PPI phenotype, which is in good accordance with results obtained in rodent schizophrenia models and humans^42–45,53,54^. Interestingly, *Nmdar1* overexpression also resulted in a PPI deficit. This suggests that deviation from an optimal range of relevant gene activity in any directions can be decremental to cognitive processes, as it is proposed by John E. Kraus^77^.

### Significance and opportunities

When attempting to introduce *Drosophila* into the candidate gene and NPD drug discovery process, its role can be best placed as the first whole animal model after *in vitro* screens or replacing them, and before rodent model tests. Both target validation and lead optimisation steps could be performed in “humanized flies”, in which the human orthologue of the fly gene can be expressed *in lieu* of the original *Drosophila* gene^78^. We propose that it is readily possible to employ various approaches to help unravelling the genetic, molecular, cellular and system biology components of NPDs in fly models.

We hope that our work can contribute to finding solutions for the treatment of such debilitating mental disorders as Schizophrenia^79^ and Bipolar Disorder^80^. It further may contribute to develop treatment for Neurodevelopmental disorders including Autistic Spectrum Disorder^81^, Attention Deficit Hyperactivity Disorder (ADHD)^82^, and monogenic conditions such as Fragile-X syndrome^83^, all known to affect PPI performance in human patients.

The applications of the *Drosophila* light-off jump response PPI paradigm can include large-scale genetic screens and efforts to identify PPI-related novel molecular/genetic components, even testing multiple combinations of mutations in mental disorder related genes. An advantage of our assay is that the light-off jump circuitry is almost completely described^74,84^ and there are tools available to target the individual components, facilitating mechanistic studies and treatment target identification.

## Methods

### Automated light-off jump response Habituation and Pre-Pulse Inhibition system

For each fly housed individually in a behaviour chamber, the jump-and-flight response (Yes or No, i.e., 1 or 0) is detected by the sound of wing vibrations using a pair of microphones. The microphone outputs are connected to a printed circuit board (PCB), which amplifies the signal and includes a noise-cancellation function. The system produces various output files providing information about the occurrence of light-off jump responses and their timing.

Eight behaviour chambers are built in a behaviour box, and two boxes form the automated high-throughput light-off jump response Habituation and Pre-Pulse Inhibition system (Aktogen Limited; see Supplementary Fig. 1). Since one experimenter can easily operate two systems at the same time, altogether 32 flies can be tested in parallel.

The chambers (made of polytetrafluoroethylene) are illuminated with green light, provided by 525 nm LED light sources. Light-off stimuli are regulated by an Intelligent Light Controller Unit (ILC box) with Arduino light controller board, ensuring high precision control of light intensity (0 to 65,535 arbitrary units) and duration (1 to 4,294,967,295 ms). Light intensity measured in lux changes linearly with settings in arbitrary units (Supplementary Fig. 4).

To ensure valid jump-and-flight response detection, each chamber is equipped with two microphones and a custom-made differential – noise cancelling – amplifier. The noise-cancelling function, in which two equal inputs (external noise) on the microphone pairs electronically cancel each other out, allows specific amplification of fruit-fly-generated sound in the behaviour chamber in the presence of external noise up to 50 dB. The 1,000x linear sound amplification across the 1-880 Hz range, combined with an experimentally defined noise threshold for jump-and-flight response ensures reliable detection of the startle response.

The amplified output sound signals are collected and analysed using custom-made software written using National Instruments LabVIEW. The LabVIEW software allows the user to set experimental parameters or choose from various optimized settings (e.g., stimulus intensity, duration, inter stimulus intervals).

### Fly stocks

Flies were maintained at 25 °C on standard cornmeal-yeast-agar medium at a 12-hours light-dark cycle. Fly husbandries were performed using standard genetic techniques. Wild-type isogenic Canton-S w^1118^ (Iso31)^85^ was from Steven Russel lab, University of Cambridge, UK, Department of Genetics. Three RNAi silencing constructs, 1. Nmdar1 RNAi, stock #37333 (w^1118^; P{GD2808}v37333), 2. stock #37334 (w^1118^; P{GD2808}v37334) and 3. dysbindin RNAi gene silencing construct, stock #106957 (w^1118^; 106957 P{KK102468}VIE-260B) were generated by Vienna Drosophila Resource Center (VDRC). The following stocks were obtained from Bloomington Drosophila Stock Center (BDSC): 1. Nmdar1 RNAi gene silencing construct generated by Transgenic RNAi Project (TRiP) stock #41666 (y1 sc* v1; P{TRiP.HMS02199}attP2), 2. deletion including Nmdar1 gene, stock#23146 (w1118; Df(3R)BSC179/TM6B, Tb1), 3. the UAS-Nmdar1 overexpression line #8275 (y, w^1118^; UAS-Nmdar1) and 4. white RNAi gene silencing construct stock# 32067 (w^+^,GMR-wIR^86^). The dysbindin^dsRNA-A^ RNAi gene silencing construct was generated in our laboratory (for details see below). To silence the expression of Nmdar1 in the central nervous system we employed the pan-neural driver line BDSC stock# 458 (w^−^ , elav-Gal4^c155^) as part of the UAS/Gal4 system. The w^−^, elav-Gal4^C155^, GMR-wIR recombinant line was established in our laboratory by meiotic recombination of w^−^, elav-Gal4^c155^ and w^+^, GMR-wIR on the X chromosome. Recombinants were selected according to their white eye colour and by the ability to drive the expression of UAS-GFP in the CNS.

For detailed description of genotypes in experiments see Supplementary Table 1.

### PPI behaviour experiments

The PPI experiments were performed under standardized conditions at 25°C and 70% relative humidity. To obtain experimental flies either from crosses or strains, 10-15 inseminated females were let to lay eggs for 24 hours in a bottle, and the resulted – typically male – offspring were collected by cold anaesthesia as freshly hatched (age of < 24 hours) adult flies. After reaching 5-7 days of age, the flies were placed inside the behaviour chambers and allowed to adapt for 5 minutes before the behaviour experiment was started.

Light-off or dimming stimuli evoked a startle jump response in flies. A light-off/dimming stimulus was defined in terms of its changing level of light intensity and duration. In PPI experiments the Initial light intensity was chosen to be 6.36 lux (arbitrary light setting 50; see Supplementary Fig. 4) and the following stimuli were presented to the flies (see also Fig. 1c):

- Pre-pulse (PP, 50→35 dimming, 15 ms duration),
- Pulse (P, 50→10 dimming, 15 ms duration) and
- “Pre-pulse & Pulse” combined [PPI, 50→35 & 50→10 dimming, 15 ms duration for both stimuli with different durations of Inter Pulse Intervals (IPI)].

One PPI experiment consisted of 10 repeats of PP, 10 repeats of P and typically 6×10 repeats of PPI stimuli per fly, randomly ordered. In PPI combinations the PP and P stimuli were separated by different Inter Pulse Intervals of 5,15, 30, 50, 100 and 200 ms. Each stimulus presentation was separated by a 5 s Inter Trial Interval (ITI), which ensures the subsequent trials to be completely independent of one another (see Fig. 1e).

In a typical PPI experiment different genotypes were tested on the same day. Each genotype was represented by 32 flies per experimental day. Each fly was typically exposed to 80 trials per experiment per day (lasting for about 7 minutes). As a result, in a typical 4-day or 6-day experiment the total number of stimuli presented was 320 or 480, respectively.

### Data analysis

Jump was defined as electronically amplified sound from the behaviour chamber higher in voltage than the experimentally set jump threshold. Accordingly, the PPI system generated output files containing “1” and “0” values for jump events and non-jump events, respectively (Raw jump data). From these values, Relative jump frequencies for each individual fly and stimulus type (i.e., PP, P and PPI) were determined with the help of a custom-made PPI analysis software utilizing base R functions^87^, and the canonical PPI Index^55^ was calculated according to the following formula:

$$\text{PPI}\;\text{Index}=100-\frac{\text{PPI}\;\text{score}}{\text{P}\;\text{score}}*100$$

where “PPI score” and “P score” values correspond to Relative jump frequency to PPI stimuli and Relative jump frequency to Pulse stimuli, respectively.

PPI Index=0 indicates no pre-pulse inhibition, PPI Index=100 indicates perfect pre-pulse inhibition.

Alternatively, the PP, P and PPI Relative jump frequencies were directly used in mixed-effects model analysis.

### PPI statistical analysis

The effect of genotype on Relative jumping frequencies was analysed with generalized linear mixed-effects model (GLMM) using ‘glmer’ function (with binomially distributed jump data and logit link functions) from the ‘lme4’ R package^69^.

We applied two types of models, in both of which the number of jumps within a trial follows a binomial distribution:

$$y_{i}∼Binomial\left(n_{i},p_{i}\right)$$

where *i* is the index of a given trial consisting of 10 stimuli, *y*_*i*_ is the number of jumps as a response to the stimuli in a given trial ranging between 0 and 10, *n_i_* is the number of stimuli administered (10) and *p_i_* is the probability of jumping as a response to the stimuli. Values of *p_i_*, were estimated by the models below. These are logistic regression models, meaning that *logit(p_i_)*, i.e., the log-odds of jumping is modelled as the linear combination of the predictors (e.g., Genotype). They are also mixed effect models, meaning that some of the predictors (‘fixed effects’, e.g., Genotype) are modelled as having a constant effect which is in itself of interest, while others (e.g., Fly ID) are modelled as random effects, with an average value of 0, and a variability that is not explained by the fixed effect predictors in the model, e.g., the variability from fly to fly, which follows a normal distribution around 0.

First type of model examined the effect of genotype on Relative jump frequency to Pulse or Pre-Pulse stimuli with genotype as a fixed effect. Fly ID, Day (testing day), System (behaviour system; usually two systems are used in parallel), Box (behaviour box; two boxes form a system), Chamber (behaviour chamber, houses an individual fly; 8 chambers are built in a box), Operator (experimenter) and Run number (32 experiments run simultaneously) were included as categorical random effects (Formula 1). The model estimates the difference between two genotypes as the difference in their log-odds of jumping, which is the same as the logarithm of their odds ratio. The odds of jumping are the probability of jumping divided by the probability of not jumping as a response to the given stimulus. The odds ratio for two genotypes is the ratio of their odds. An odds ratio of 1 means equal probability of jumping, >1 means higher probability of jumping for the genotype in the numerator.

Formula 1): $$logit\left(p_{i}\right)=\beta_{0}+\beta^{Genotype}\;\cdot \;Genotype_{i}+\alpha_{k[i]}^{Run}+\alpha_{l[i]}^{Chamber}+\alpha_{m[i]}^{Operator}+\alpha_{n[i]}^{Fly\;ID}$$

$$\alpha_{k}^{Run}∼N\left(\alpha_{p[k]}^{Day},\sigma_{Run}^{2}\right)$$

$$\alpha_{p}^{Day}∼N\left(0,\sigma_{Day}^{2}\right)$$

$$\alpha_{l}^{Chamber}∼N\left(\alpha_{q[l]}^{Box},\sigma_{Chamber}^{2}\right)$$

$$\alpha_{q}^{Box}∼N\left(\alpha_{r[q]}^{System},\sigma_{Box}^{2}\right)$$

$$\alpha_{q}^{System}∼N\left(0,\sigma_{System}^{2}\right)$$

$$\alpha_{m}^{Operator}∼N\left(0,\sigma_{Operator}^{2}\right)$$

$$\alpha_{n}^{Fly\;ID}∼N\left(0,\sigma_{Fly\;ID}^{2}\right)$$

The other model examined the effect of genotype on PPI, i.e., suppression of Relative jump frequency to Pulse stimuli when these are preceded by Pre-Pulse stimuli at all tested IPIs (IPI: 5, 15, 30, 50, 100, 200, 300 ms). Genotype, IPI, jump response to Pulse (natural logarithm of the relative jumping frequency plus 0.1 to avoid taking the logarithm of 0), interaction between jump response to Pulse stimuli and IPI, and interaction between genotype and IPI were treated as fixed effects. Fly ID, Day, System, Box, Chamber, Operator and Run were treated as random effects (Formula 2). For the categorical variable ‘IPI’, a reference category was added representing Pulse stimuli alone (without Pre-Pulse). As a result, the interaction term ‘Genotype * IPI’ is a log odds-ratio estimating the additional effect of a given genotype on the log-odds of jumping when both Pulse and Pre-Pulse stimuli are applied (with a particular IPI) compared to the effect of the same genotype when only Pulse stimuli are applied. In other words, for a given IPI it represents the difference between two genotypes in their degree of PPI (i.e., in the effect of Pre-Pulse preceding Pulse stimuli). An odds ratio < 1 indicates a lower chance for jumping in the case of adding PPI stimulus compared to Pulse alone, implying stronger Pre-Pulse Inhibition for the genotype in the numerator.

Formula 2): $$logit\left(p_{i}\right)=\beta_{0}+\beta^{Genotype}\;\cdot \;{\mathrm{Genotype}}_{i}+\beta^{IPI}\;\cdot \;IPI_{i}+\beta^{Genotype\;\cdot IPI}\;\cdot \;{\mathrm{Genotype}}_{i}\cdot IPI_{i}+\beta^{\mathrm{Pulse}}\cdot {\mathrm{Pulse}}_{n[i]}+\beta^{\mathrm{Pulse}\cdot IPI}\cdot {\mathrm{Pulse}}_{n[i]}\cdot IPI_{i}+\alpha_{k[i]}^{Run}+\alpha_{l[i]}^{Chamber}+\alpha_{m[i]}^{Operator}+\alpha_{n[i]}^{Fly\;ID}$$

, where

Pulse_n_ = ln(0.1 + relative frequency of jumping to pulse for fly*n*), e.g., if fly n jumps 8 out of 10 times, ln(0.1 + 0.8)

### Molecular cloning of dysbindin RNAi construct

A 585 bp long region (3 L: 18,868,047... 18,868,631; FB2022 release=r6.46) of the first exon of *Dysbindin* gene was PCR amplified with forward (5’ cagaTCTGTCGTCCAGCAGGAGCAGTAG 3’) and reverse (5’ cgtcgaCGCTGTTTGTACTCCTCCATATCC 3’) primers carrying a BglII and a SalI site at their 5’ ends, respectively. The resulting PCR product was inserted into the PCR cloning vector Topo-TA (Thermo Fisher Scientific, #450641) and subsequently transferred as BglII, SalI double digested fragment into BglII, XhoI sites of pWizMod[vector (GenBank accession number AB186054)^88^. In an additional cloning step, the same insert was cut out from Topo-TA by KpnI, XbaI double digestion and inserted into the KpnI, XbaI sites of the intermediate plasmid, resulting in *pDysbindin* ^*dsRNA-A*^ with the desired inverted repeat arrangement. The plasmid was microinjected into *w*^*1118*^*; Sb, Δ2-3/TM6* embryos, and P-transposase-based integrants were selected for their mini-w^+^ eye colour.

### RNA preparation and Quantitative Real-Time PCR (qRT-PCR)

Total RNA from twenty *Drosophila* heads for each genetic combination was purified using the RNA isolation kit of Macherey-Nagel (Macherey-Nagel, Duren, Germany). All the preparation steps were carried out according to the manufacturer’s instructions. RNA samples were stored at −80°C in the presence of 30 U RiboLock RNase Inhibitor (Thermo Fisher, Waltham, MA, USA) for further analysis. The quantity of isolated RNA samples was checked by NanoDrop 3.1.0 (Thermo Fisher, Waltham, MA, USA).

1 μg of total RNA was reverse transcribed using the High-Capacity cDNA Archive Kit (Thermo Fisher Scientific, Waltham, MA, USA) according to the manufacturer’s instructions in a T100 Thermal Cycler machine (BioRad, Hercules, CA, USA). Briefly, the reaction mixture was the following: 10 μl (1μg) total RNA template, 2 μl 10x RT Buffer, 0.8 μl dNTP, 2 μl Random primers and 1μl Reverse transcriptase in 20 μl final volume. The temperature profile of the reverse transcription was the following: 10 min. at room temperature, 2 hours at 37 °C, 5 min. on ice and finally 10 min. at 75 °C for enzyme inactivation.

After two times dilution, 1 μl of the diluted reaction mix was used as template in the QRT-PCR. The reaction was performed on a RotorGene 3000 instrument (Qiagen, Hilden, Germany) with gene-specific primers and qPCRBIO SyGreen Mix Lo-ROX mix (PCR Biosystems, London, UK) according to the manufacturer’s instructions at a final primer concentration of 250 nM under the following conditions: 2 min. at 95°C, 40 cycles of 95°C for 5 sec., 60°C for 30 sec. Melting temperature analysis was done after each reaction to check the quality of the products. Primers were designed online using the Roche Universal Probe Library Assay Design Center or the Integrated DNA Technologies qPCR Assay Design RealTime PCR Tool. The quality of the primers was verified by MS analysis provided by Bioneer (Daejeon, South Korea). Individual threshold cycle (Ct) values were normalized to the mean Ct values of *DmRap, DmGiant, DmMnf* internal control genes. Relative gene expression levels are presented as percentage to wild type, calculated using the formula (fold change=2^ΔΔCt^), according to the ΔΔCt method^89^. Information about the genes and the primers is summarized in Supplementary Table 4.

### Statistical analysis of qRT-PCR data

RNA samples were prepared and tested in four parallels (n=4) for each genetic combination. Statistical comparison of normalized Ct (ΔCt) values of control and mutant genotypes was done by Student’s t-test (two-tailed, unequal variance). Results were summarized in Supplementary Table 3.
